# Acquisition of naturally occurring antibody responses to recombinant protein domains of *Plasmodium falciparum *erythrocyte membrane protein 1

**DOI:** 10.1186/1475-2875-7-155

**Published:** 2008-08-16

**Authors:** Claire L Mackintosh, Zoe Christodoulou, Tabitha W Mwangi, Moses Kortok, Robert Pinches, Thomas N Williams, Kevin Marsh, Christopher I Newbold

**Affiliations:** 1 Kenya Medical Research Institute Centre for Geographic Medicine Research Coast (KEMRI-CGMRC), Kilifi District Hospital, Kilifi, Kenya; 2 Molecular Parasitology Group, Weatherall Institute of Molecular Medicine, John Radcliffe Hospital, Oxford, UK; 3 Nuffield Department of Medicine, John Radcliffe Hospital, Oxford, UK; 4 Department of Paediatrics, John Radcliffe Hospital, Oxford, UK

## Abstract

**Background:**

Antibodies targeting variant antigens expressed on the surface of *Plasmodium falciparum *infected erythrocytes have been associated with protection from clinical malaria. The precise target for these antibodies is unknown. The best characterized and most likely target is the erythrocyte surface-expressed variant protein family *Plasmodium falciparum *erythrocyte membrane protein 1 (*Pf*EMP1).

**Methods:**

Using recombinant proteins corresponding to five domains of the expressed A4 *var *gene, A4 *Pf*EMP1, the naturally occurring antibody response was assessed, by ELISA, to each domain in serum samples obtained from individuals resident in two communities of differing malaria transmission intensity on the Kenyan coast. Using flow cytometry, the correlation in individual responses to each domain with responses to intact *A4-*infected erythrocytes expressing A4 *Pf*EMP1 on their surface as well as responses to two alternative parasite clones and one clinical isolate was assessed.

**Results:**

Marked variability in the prevalence of responses between each domain and between each transmission area was observed, as wasa strong correlation between age and reactivity with some but not all domains. Individual responses to each domain varied strikingly, with some individuals showing reactivity to all domains and others with no reactivity to any, this was apparent at all age groups. Evidence for possible cross-reactivity in responses to the domain DBL4γ was found.

**Conclusion:**

Individuals acquire antibodies to surface expressed domains of a highly variant protein. The finding of potential cross-reactivity in responses to one of these domains is an important initial finding in the consideration of potential vaccine targets.

## Background

Maintaining *Plasmodium falciparum *infections whilst limiting morbidity and mortality is a feature of the non-sterile immunity acquired by individuals living in malaria endemic areas. Studies whereby antibodies were passively transferred from immune to non-immune individuals suggested this immunity is, at least in part, antibody-mediated [1,2]. Humans exposed to malaria can mount an antibody response to many parasite antigens including those present on the sporozoite, merozoite and those on the surface of the infected erythrocyte [3-5]. Parasite induced antigens on the infected red cell surface are potentially important targets for protective immunity because they are exposed for long periods of the erythrocytic cycle and serve critical biological functions [6]. Following infection children mount antibodies directed against the infected erythrocyte surface, specific to the infecting isolate [7-10] and such antibodies are associated with protection from subsequent clinical malaria with the homologous parasite [8].

The most extensively characterized of the proteins expressed at the infected red cell surface are the products of the *var *genes, *Plasmodium falciparum *erythrocyte membrane protein 1, (*Pf*EMP1) [11]. *Pf*EMP1 is a family of extracellular, highly polymorphic and clonally variant adhesion molecules [12-16]. They are expressed on the surface of the red cell at around 18 hours after invasion and remain present throughout the second half of the intra-erythrocytic cycle [12]. They exhibit a domain structure and the domains bear homology to the cysteine-rich binding domains of varied Plasmodium molecules involved in the binding to and invasion of erythrocytes; EBA-175, the *P. falciparum *glycophorin A receptor and the *Plasmodium vivax *and *Plasmodium knowlesi *ligands that allow invasion of Duffy blood-group positive erythrocytes [17-19]. These domains are called Duffy-binding like domains (DBL) and they are interspersed with regions containing multiple cysteine residues termed the cysteine-rich interdomain regions (CIDR).

Using a panel of recombinant proteins corresponding to the domains of one particular *Pf*EMP1 protein, A4 *Pf*EMP1 from the A4 laboratory parasite line, domain-specific antibodies prior to the transmission season in two communities in Kenya with differing transmission characteristics were measured. The presence of these antibodies was related to the likelihood of experiencing clinical malaria during the subsequent transmission season. It was shown that humans are capable of mounting anti-*Pf*EMP1 domain-specific antibodies and that the prevalence of these antibodies is related to exposure. Furthermore responses to one recombinant domain, DBL4γ, show evidence of cross-reactivity or perhaps are being directed at a more conserved epitope.

## Materials and methods

### Study population

This work was carried out at the Kenya Medical Research Institute, Centre for Geographic Medicine Research Coast situated at Kilifi District Hospital, 50 km north of Mombasa on the coast of Kenya. The hospital serves around 240,000 people living north and south of an ocean creek. Individuals investigated during these immuno-epidemiological studies were resident in two sites in Kilifi District (within 20 km of each other), Chonyi and Ngerenya. These study sites have been described in detail elsewhere [20]. Inhabitants of these areas are predominantly Mijikenda, sharing similar beliefs and customs. Residents of Ngerenya receive, on average 10 infective bites/person/year, [21], whereas residents of Chonyi have an estimated 50 bites/person/year [22]. The annual incidence of clinical malaria among the two communities varied with both age and area. In children less than one year of age, the incidence was higher in Chonyi than in Ngerenya (IRR 1.56 [95% CI 1.18–2.06]p = 0.002), there was no difference between the two areas in children aged 1–3 years and in those aged 4 – 19 years the incidence was higher in Ngerenya (IRR 0.45 [95% CI 0.4–0.51]p = 0.001) [20].

### Sample collection

Sera collection and active surveillance were conducted as part of a study examining the clinical epidemiology of malaria under differing transmission conditions[20]. In brief, serum was collected in October 2000 from 1,222 individuals resident in both areas aged between six months and 85 years. A blood slide was prepared for every individual in the cohort at the time of serum collection in order to define their pre-clinical surveillance status as parasite positive or negative. All individuals included in this study were asymptomatic and afebrile at the time of cross-sectional survey. The cohorts were then followed for evidence of malaria by weekly active surveillance for fever. Malaria was defined as a febrile episode with an axillary temperature greater than 37.5°C and a parasitaemia of greater than 2500 parasites/μl above one year of age, and fever plus any parasitaemia below a year. These have been determined to be sensitive and specific malaria case-definitions in both study communities[20]. Twenty non-malaria exposed control sera were collected from Oxford, UK.

### Parasites

These experiments were performed using a laboratory clone of parasite, A4. This particular parasite was chosen for a number of reasons. There exists a monoclonal antibody, BC6, which allows *in vitro *selection of A4 parasites expressing specifically A4 *Pf*EMP1[23]. A4 *Pf*EMP1 has a well-described and classified sequence already published with established domain boundaries and it displays a common cytoadherent phenotype, binding to both CD36 and ICAM-1 [24].

The parasite clone A4 having undergone prior selection with BC6 is denoted A4U. In addition two further laboratory clones, A4 40-cycle (parasites of the A4 lineage which have been left in culture for 40 cycles with no selection) and 3D7, and one clinical isolate, P1, obtained from a five year old child admitted to Kilifi District Hospital with moderately severe malaria were used to investigate antibody reactivity against the intact infected erythrocyte by flow cytometry. Using the monoclonal antibody BC6, flow cytometry demonstrated expression of the A4 *var *gene of between 88–95% in A4U parasites and between 11 and 25% in A4-40 cycle parasites. There was no recognition of either 3D7 or P1 with BC6.

### Expression of A4 PfEMP1 domains; DBL1α, DBL2β, CIDR1α, DBL4γ, DBL5β in BL21-CodonPlus-Ril *E. coli*

For secreted, IPTG-inducible expression in BL21-CodonPlus-Ril *Escherichia coli *(Stratagene UK), vector pMal-c2x (New England Biolabs) was used. Primers for PCR amplification were as follows (Accession number L42244): DBL1α forward 5'-CATGGTAGGGAGGATCCT, reverse 5'-CCCCAAGCTTGCCTATTCCGTATGAGAAAATG including the restrictions sites SmaI and HindIII respectively; CIDR1α forward 5'-TCCCCGGGGCAGGTGGATTATGTATATTCG, reverse 5'-CCCCTGCAGCTATGAATCACCAATAGCATTGG including restriction sites SmaI and PstI respectively; DBL2β forward 5'-CTCCCCGGGACGAACCAATATTCCAATGC, reverse 5'-CCTCTAGACTAGCACACATCCAACTTGGTGTC including restriction sites SmaI and XbaI respectively; DBL4γ forward 5'-GCTCCCCGGGTGCAATACAAAATATTATCCAAC, reverse 5'-CCCGCAAGCTTGCTACGAAGCAAATGTACTGTC including restriction sites SmaI and XbaI respectively; DBL5β forward 5'-GCTCCCCGGGGCTTCGAATTGTGAAC, reverse 5'-CCTCTAGACTAGATTTCGGATCGTTATTACTCG. (Domains DBL3δ and CIDR2 of the A4 *var *gene were unable to be satisfactorily cloned despite many attempts). Amino acid numbering of each recombinant domain: DBL1α 81–480, CIDR1α 590–841, DBL2β 832–1229, DBL4γ 2006–2437, DBL5β 2435–2802. PCR fragments corresponding to each domain were purified using a QIAquick PCR purification kit (Qiagen, Hilden, Germany) and were ligated into pCR 2.1 vector (Invitrogen), containing the α-peptide of b-galactosidase to allow blue-white selection in the presence of 5-bromo-4-chloro-3-indolyl-b-D-galactoside (X-Gal). Sub-cloning efficiency cells DH5α were transformed and positive recombinant clones were identified by loss of α-complementation. Transformants were checked, by PCR performed with vector-specific primers, for the presence of domain-specific DNA and the plasmid DNA was purified using Wizard Plus SV Minipreps (Qiagen, Hilden, Germany). Purified pCR 2.1 plasmids containing individual domains were digested with the correct restriction enzymes and ligated into pMal-c2x vector (New England Biolabs), which had been previously cut appropriately. DH5α cells were again transformed and blue-white screening as before identified positive recombinant clones. DNA sequencing using an ABI 373 Prism automated sequencing system with a Big Dye terminator sequencing kit (Applied Biosystems, Foster City, California) determined nucleotide sequences of the inserts. Sequence identities were confirmed by BLAST analysis. For protein expression BL21-CodonPlus-Ril competent cells (Stratagene) were used. The transformed cells were grown in Luria-Bertani medium with 0.2% glucose, ampicillin (50 μg/mL) to maintain the expression plasmid and chloramphenicol (50 μg/mL), to an optical density at 600 nm (OD_600_) of 0.3 to 0.5 at temperatures between 25°C and 37°C depending on the individual construct. Each culture was then induced to express the MBP-DBL/CIDR fusion proteins in the presence of 0.1 mM isopropyl-β-D-thiogalactoside (IPTG). The samples were spun and the pellet re-suspended in 1 M Tris lysis buffer. The cells were disrupted by sonication (12 pulses × 1 min). After spinning each MBP-fusion protein was purified by chromatography on amylose resin as recommended by the manufacturer (New England Biolabs), using 100 mM maltose for elution. After purification, the protein obtained was stored in the following buffer: 10 mM Tris-HCl, pH 7.4, 0.2 M NaCl, 10 mM β-mercaptoethanol, 1 mM EDTA at -80°C. Protein purity was determined by sodium dodecyl sulphate-polyacrylamide gel electrophoresis with 10% polyacrylamide and quantity was estimated by a protein assay kit (Bio-Rad, Munich, Germany) as recommended by the manufacturer.

### Enzyme-linked immunosorbant assay (ELISA)

After checkerboard assays to determine the optimal coating concentration, 200 ng of DBL2β and DBL4γ and 400 ng of DBL1α, CIDR1α and DBL5β were coated onto individual wells in Nunc transparent, flat-bottomed 96 well plates (Nunc Technology) in 100 μl phosphate buffered saline (PBS). An equivalent molar concentration of MBP alone was coated as a control. In addition schizont extract of the A4 strain of *Plasmodium falciparum *was produced and coated onto plates in PBS using the methods described by Ndungu *et al *[25]. The plates were incubated overnight at 4°C, washed in PBS with 0.05% Tween 20 and blocked with 200 μl blocking buffer (10% skimmed milk in PBS with 0.5% Tween 20) for 1 hour at 37°C. The plates were washed again as before and 100 μl of human sera (diluted 1:100 with blocking buffer) was incubated in duplicate for 1 hour at 37°C. The wells were again washed and 100 μl of HRP-conjugated rabbit anti-human IgG (Dako Ltd.), at a dilution of 1:5000 was added and the plates incubated again for 1 hour at 37°C. Detection was by the addition of O-phenylenediamine/H_2_O_2 _(Sigma) for 15 minutes in darkness. The mean OD value, taken at 492 nm, was calculated for each sample after correction for binding to MBP alone. A serum was scored as positive if the corrected OD value was higher than the mean + 3 SD of 20 negative control sera from UK residents who had never been exposed to malaria. All sera were screened in duplicate and a selection repeated at a later date. OD values were standardized against a pre-screened high positive standard on each plate. Please see additional files 1 and 2 for the mean OD values for the positive standards on each plate and for the range of OD values obtained with sera from 20 non-malaria exposed UK donors respectively.

### Flow cytometry

Using parasite clones A4U, A4-40 cycle and 3D7 and the clinical isolate, denoted P1, cryo-preserved trophozoite-infected erythrocytes at between 1 and 5% parasitaemia were thawed through the sequential restoration of isotonicity [26]. They were washed twice in RPMI and the pellet was resuspended at 1% haematocrit in 0.1% bovine serum albumin/phosphate-buffered saline (0.1%BSA/PBS). 1 μl of human serum was pipetted into separate wells of a 96-well U-bottomed plate (Nunc Technology) and 9 μl of the infected erythrocyte cell suspension was added to each well giving a final test serum concentration of 1:10. The reaction mixture was incubated at room temperature for 1 hour, following which the cells were spin washed three times with 0.1%BSA/PBS. The cells were then resuspended in 25 μl 0.1%BSA/PBS containing the secondary antibody, rabbit anti-human IgG at a concentration of 1:100. Again the reaction mixture was incubated for 1 hour at room temperature after which a further three washes were performed as before. Finally 25 μl of 0.1%BSA/PBS containing a 1:100 dilution of swine anti-rabbit IgG coupled to FITC and 10 μg/ml of ethidium bromide was added to each well. A further incubation at room temperature in darkness, for 1 hour was done after which, following a further series of washes; at least 1,000 infected erythrocytes were counted on an EPIC/XL flow cytometer (Coulter-electronics, UK).

Reactivity against the infected erythrocyte surface was scored as mean fluorescent intensity using the method of Williams *et al *[27]. In detail, mean fluorescence of parasite-infected erythrocytes was determined using the formula:

**(d-c)-(b-a)**

**a **= the mean fluorescence intensity (MFI) of uninfected erythrocytes following incubation in negative control plasma; **b **= MFI parasitized erythrocytes following incubation in negative control plasma; **c **= MFI uninfected cells incubated in immune plasma or test antibody and **d **= MFI parasitized erythrocytes incubated in immune plasma or test antibody.

### Statistical analysis

All statistical analyses were performed using Stata version 8 (Statacorp, TX, USA). To investigate the relationship between parasite status at the time of cross-sectional bleed and subsequent antibody levels, a multiple linear regression model, controlled for age, expressed as a factor of 6 months duration, location and exposure (estimated by responses to schizont extract) was performed. The results are expressed as coefficients describing the difference in antibody levels attributable to parasites.

Significant differences in two or more continuous variables were calculated using the Wilcoxon rank sum test. The Chi-squared test for trend was used to assess a trend across groups.

In a randomly selected subgroup of individuals (148 from Chonyi and 142 from Ngerenya, all ages represented), total IgG responses to erythrocytes parasitized with A4U, A4-40 cycle, 3D7 and P1 were also measured using flow cytometry. The correlation of responses to each individual domain with responses to the parasitized erythrocyte surface was assessed using the Spearman rank correlation coefficient.

In order to investigate any association of domain-specific antibody responses with protection from clinical malaria, initially the univariate association between detectable serum IgG to each antigen (measured as OD), and whether or not the individual had an episode of clinical malaria during the subsequent six months was investigated by fitting the continuous variable against a binomial distributed outcome using logistic regression. As antibody reactivity is known to increase with exposure, a multiple logistic regression was then performed with age, location and reactivity to schizont extract, as a marker of exposure, included as co-dependent variables. Results are expressed as odds ratios per 10 fold increase in antibody levels.

## Results

### Naturally occurring antibody responses to recombinant PfEMP1 domains

**Figure 1:**
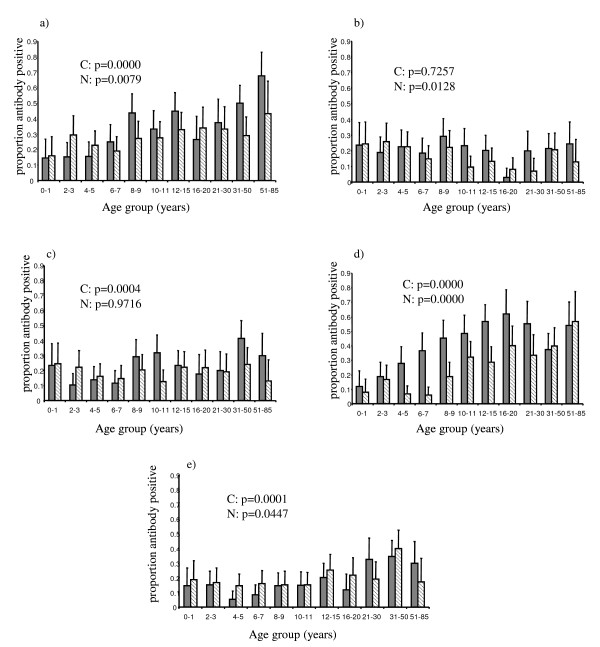
**Proportion of individuals in each age group recognizing recombinant domains**. Sera from 1222 individuals older than six months, was tested for reactivity against each recombinant protein in turn using ELISA. Responses were scored as positive if the mean OD obtained was greater than the mean OD plus 3 standard deviations of a panel of 20 non-malaria exposed donors. The solid grey bars refer to individuals resident in Chonyi and the hatched bars to individuals resident in Ngerenya. a) DBL1α, b) CIDR1α, c) DBL2β, d) DBL4γ, e) DBL5β. P-values given are chi-squared for trend, C = Chonyi N = Ngerenya.

Evidence for age-associated acquisition of domain-specific IgG was apparent for responses against recombinant domains DBL1α and DBL4γ in both Ngerenya and Chonyi (Figure 1). No appreciable increase in antibody acquisition with age was seen with CIDR1α, in either area and with responses to both DBL2β and DBL5β, the trend for increasing acquisition of responses with age was significant in the higher of the two transmission areas, Chonyi

Noticeable differences could be seen in the response to the different *Pf*EMP1 domains. For example it is clear that a greater proportion of individuals recognize DBL1α and DBL4γ than the other three domains with overall 30.8% (95% CI 26.2 – 35.8%) and 32.5% (95% CI 27.9 – 37.3%) of individuals recognizing DBL1α and DBL4γ respectively, compared to 18.8% (95% CI 13.9 – 24.3%), 21.2% (95% CI 16.4 – 26.7%) and 19.6% (95% CI 14.7 – 25.2%) for CIDR1α, DBL2β and DBL5β respectively (p < 0.0001 for comparison of overall recognition of DBL1α or DBL4γ with any other domain Wilcoxon ranksum).

There was a clear increase in proportion of responders and rate of acquisition of responses in Chonyi compared to Ngerenya. For example with DBL4γ the maximum response in Chonyi occurs in the 16 – 20 year age group with 61.8% (95% CI 43.6 – 77.8%) of individuals in this age group recognizing this domain. This is compared to the maximum response in Ngerenya against the same domain, which did not occur until the 51 – 85 year age group and was lower at 56.5% (95% CI 34.5 – 76.8%). Acquisition of antibody responses against DBL4γ was more rapid in Chonyi where, by the age of 8 – 9 years 45.2% (95% CI 32.5 – 58.3%) of children responded compared to 18.6% (95% CI 9.70 – 30.9%) of children resident in Ngerenya (p = 0.0019 Wilcoxon ranksum). For responses against DBL1α, the situation was different with acquisition in both areas continuing throughout adulthood. The maximum response in both areas did not occur until the 51 – 85 year age group although as with DBL4γ, the maximum response was greater in Chonyi, 67.6% (95% CI 50.2 – 81.9%) than in Ngerenya at 43.5% (95% CI 23.2 – 65.5%) although this difference was not significant. Overall a greater proportion of individuals resident in Chonyi showed evidence of reactivity against DBL1α and DBL4γ but not CIDR1α, DBL2β or DBL5β. The two-sample Wilcoxon rank sum for the differences in response between the two locations gave the following significance values: DBL1α p = 0.0128, CIDR1α p = 0.0594, DBL2β p = 0.0774, DBL4γ p = 0.0000 and DBL5β p = 0.3848.

### Individual variation in antibody responses to recombinant domains of A4-PfEMP1

461 individuals recognized none of the domains tested and of these 57.7% were resident in Ngerenya and 42.3% in Chonyi. Of these 461 individuals, 61.2% were aged less than 10 yrs. 350 individuals only recognized 1 domain to the exclusion of all the others, 160 of these individuals (45.7%) were aged less than 10 years. Of those only recognizing one domain, 26.6% recognized DBL1α only, 11.7% recognized DBL2β, 12.7% recognized CIDR1α, 37.4% DBL4γ only and 11.4% DBL5β only, making DBL4γ significantly more likely to be the single domain recognized (OR 1.67 (95% confidence interval 1.24 – 2.24) p = 0.001). Only 38 individuals recognized all 5 domains tested (3.12% of the whole cohort), of these 24 (63.2%) were aged over 10 yrs.

**Figure 2:**
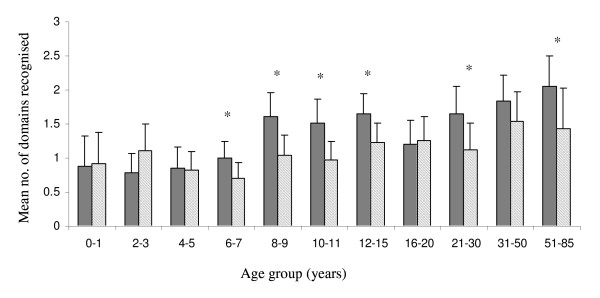
**Mean number of domains recognised with age**. Graph shows mean number of domains, plus upper 95% confidence limits, recognized by individuals in each age group. The dark grey bars refer to individuals in Chonyi and the hatched bars to individuals resident in Ngerenya. *=*P*-value <0.05 (Wilcoxon Ranksum).

Overall the number of domains recognized increased with age in both areas (Figure 2). In six of the age groups, individuals resident in Chonyi recognized a significantly greater number of domains.

### Association of asymptomatic parasitaemia and anti-A4PfEMP1 antibody responses

It has previously been noted that antibody responses to a number of parasite antigens are enhanced in younger individuals who are parasitized at the time of blood sampling [28-30]. Using a logistic regression analysis taking into account age, exposure (as estimated by responses to whole schizont extract) and location, and restricting the analysis to those less than 10 years, individuals with parasites in their blood at the time of bleed were significantly more likely to recognize any recombinant domain compared to recognizing no domains (OR 1.94 (95% CI 1.34 – 2.83) p < 0.001). Furthermore, using an ordered logistic regression, with the same confounding variables, those children parasite positive at the time of cross-sectional bleed were more likely to recognize a greater number of domains (OR 1.66 (95% CI 1.17 – 2.34) p = 0.004).

Using a multiple linear regression model, the effect of asymptomatic parasitaemia on the OD value obtained for each domain after controlling for age, location and exposure was determined. Having detectable parasites at cross-sectional bleed was associated with significantly higher OD values for domains DBL1α, DBL2β and CIDR1α (Table 1). There was no effect on antibody reactivity to DBL4γ or DBL5β.

**Table 1:** Effect of parasite status on antibody levels to each recombinant domain

**Domain**	**coefficient^1^**	**95% C.I.**	***P*-value**
DBL1α	0.067	0.027 – 0.117	0.001
CIDR1α	0.044	0.015 – 0.072	0.003
DBL2β	0.074	0.029 – 0.118	0.001
DBL4γ	-0.036	-0.078 – 0.006	0.1
DBL5β	0.002	-0.034 – 0.039	0.886

^1^Results from a multiple linear regression giving the coefficients for the effect of being parasite positive on the OD obtained to each domain, controlling for the confounding effects of age, location and exposure (as estimated by responses to schizont extract).

### Correlation of responses to recombinant domains with responses to the surface of erythrocytes infected with A4 parasites selected to express A4-PfEMP1

**Figure 3:**
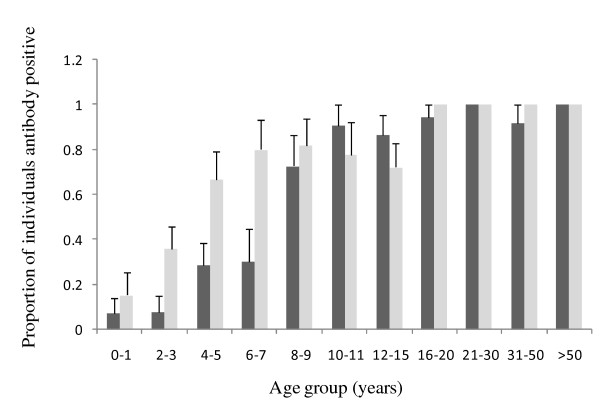
**Proportion of individuals in each age group recognizing parasite line A4u**. Sera from 272 individuals older than six months, was tested for reactivity against the parasite line A4U using flow cytometry. The proportion of individuals in each age category, with upper 95% confidence interval, scoring positive for antibody recognition are shown. Positivity was scored as defined in the text. The light grey bars represent individuals resident in Chonyi and the dark grey bars represent individuals resident in Ngerenya.

Figure 3 outlines the prevalence of responses to A4U measured by flow cytometry. Acquisition of responses with age is demonstrated. Using Spearman's rank correlation coefficient to assess significance, only responses to DBL1α and DBL4γ were positively correlated with responses to the surface of erythrocytes infected with A4 parasites selected to express A4-*Pf*EMP1, A4U (DBL1α v A4u Spearman's rho = 0.1263 *P *= 0.0315; DBL2β v A4u Spearman's rho = 0.0462 *P *= 0.4331; CIDR1α v A4u Spearman's rho = -0.1281 *P *= 0.0292; DBL4γ v A4u Spearman's rho = 0.2866 P < 0.0001; DBL5β v A4u Spearman's rho = 0.0376 *P *= 0.5239) In an attempt to control for exposure accounting for any positive correlation, a multiple linear regression model was performed with age, location, parasite status and exposure (estimated by responses to schizont extract) as independent variables, for each of the pairs. Using this model, only the correlation between responses to DBL4γ and A4u reached significance (Coefficient 0.332 (95% CI 0.101 – 0.563) *P *= 0.005).

In addition individual responses to each recombinant A4-*Pf*EMP1 domain were correlated against antibody responses to two further parasite clones and one clinical isolate as measured by flow cytometry; A4-40 cycle, 3D7 and a clinical isolate P1. Additional file 3 outlines the prevalence of responses to each of these parasites as measured by flow cytometry. Responses to DBL4γ recombinant protein correlated significantly with responses to erythrocytes infected with each of the parasites tested (DBL4γ v A4-40cycle Spearman's rho = 0.3909 p < 0.001; DBL4γ v P1 Spearman's rho = 0.3487 p < 0.001; DBL4γ v 3D7 Spearman's rho = 0.4054 p < 0.001). These correlations were maintained after accounting for previous exposure in a multiple linear regression model. There were no correlations observed between individual antibody responses measured against any other recombinant domain and any of the parasite lines tested.

### Association of anti-A4PfEMP1 antibody responses and protection from clinical malaria

When antibody positivity to the *Pf*EMP1 recombinant domains was analysed as a whole within Chonyi, no association was found between antibody response and subsequent disease experience (Table 2). When individuals were categorized by parasite status at the time of bleed, a positive association between the presence of anti-DBL1α antibodies and protection from subsequent clinical malaria was found in those parasite negative at the time of bleed. No association with protection from or susceptibility to malaria was observed with antibodies against any other recombinant domain in Chonyi and antibody responses to none of the domains were associated with protection in Ngerenya.

**Table 2:** Association of serum IgG levels to recombinant domains of *Pf*EMP1 with clinical malaria in Chonyi, Kenya for the period October 2000 until March 2001

**Chonyi**		**Slide all**	**n = 596**
Antigen	Odds Ratio	p-value	95% C. I.

DBL1α	0.75	0.580	0.26 – 2.11
DBL2β	0.63	0.472	0.18 – 2.21
CIDR1α	0.71	0.365	0.33 – 1.50
DBL4γ	0.89	0.791	0.37 – 2.13
DBL5β	0.72	0.517	0.26 – 1.95

**Chonyi**		**Slide positive**	**n = 197**

Antigen	Odds Ratio	p-value	95% C. I.

DBL1α	0.75	0.580	0.26 – 2.11
DBL2β	0.63	0.472	0.18 – 2.21
CIDR1α	0.71	0.365	0.33 – 1.50
DBL4γ	0.89	0.791	0.37 – 2.13
DBL5β	0.72	0.517	0.26 – 1.95

**Chonyi**		**Slide negative**	**n = 399**

Antigen	Odds Ratio	p-value	95% C. I.

DBL1α	0.05	0.020	0.04 – 0.63
DBL2β	0.26	0.208	0.32 – 2.10
CIDR1α	0.72	0.553	0.25 – 2.10
DBL4γ	0.49	0.290	0.14 – 1.81
DBL5β	0.49	0.343	0.11 – 2.11

^1^Results from a multiple logistic regression giving the odds ratio for becoming a case of clinical malaria in the subsequent six months if scored as antibody positive against each domain in turn controlling for for age (categorized as a factor of six months duration) and exposure (estimated by responses to whole schizont extract). An individual was deemed antibody positive if the OD obtained was greater than the mean OD plus 3 standard deviations obtained from a panel of non-exposed donors.

## Discussion

*Pf*EMP1 is currently the most plausible and best characterized of the erythrocyte surface-expressed parasite-induced proteins proposed as targets for naturally acquired protective immune responses [31]. *Pf*EMP1 is a large, diverse and structurally complex molecule. It is composed of an intracellular, highly conserved domain and a large, extracellular variant domain. Although this extracellular domain is highly polymorphic, *Pf*EMP1 variants share an overall common structure.

It has been hypothesized that naturally acquired immunity develops through the piecemeal acquisition of a large repertoire of antibodies directed against variants of this protein [8]. Supporting this possibility are experimental vaccination of aotus monkeys with selected domains from specific *Pf*EMP1 variants resulting in protection against the homologous genotype, [32], and more recent data showing cross-protection against heterologous strains of parasite after immunization with a DBL1α domain [33]. Understanding the naturally occurring antibody response to the DBL and CIDR domains making up the extracellular component of a specific *Pf*EMP1 protein is thus an important part of evaluating the usefulness of *Pf*EMP1 as a vaccine candidate.

Recent publications exploring the dynamics of the naturally acquired antibody response to *Pf*EMP1 have shown that whilst cross-reactive antibody responses to *Pf*EMP1 molecules encoded by different parasite genomes must exist; children in endemic areas acquire surface reactive antibodies to most variant surface antigens [8], and these have been associated with protection [34], there is little evidence of any cross-reactivity in responses to *Pf*EMP1 domains within a single genome [35]. Looking at responses to specific domains, Lusingu et al found evidence of protection associated with the presence of antibodies directed against a recombinant CIDR1α expressed from a large complex *var *gene implicated in severe malaria [36]. A further study has shown that antibodies directed against recombinant domains from *Pf*EMP1expressed from an isogenic parasite line of 3D7 increase both with intensity and age and that specifically, increased levels of anti-CIDR2β are associated with less clinical malaria [37]. In addition, Oguariri and colleagues compared the prevalence of antibodies directed against recombinant DBL1α domains from nine wild isolates in the sera of children and adults resident in an area hyperendemic for *P. falciparum *transmission. There was strong correlation between the age of the patients and reactivity against the recombinant DBL1α domains[38]. Here, through the use of recombinant protein technology, the naturally acquired antibody response to the extracellular domains of A4 *Pf*EMP1 in the sera of individuals resident in two areas endemic for *P. falciparum *malaria but with different transmission characteristics have been evaluated.

It is clear that there are differences in the total acquisition of domain-specific antibodies and the time over which such responses are acquired both between areas and between domains. Previous studies have described an increased prevalence of antibodies to blood-stage antigens amongst individuals with detectable parasites at the time of bleed [28-30]. A similar result was obtained in this study with regard to responses to DBL1α, DBL2β and CIDR1α, but not to DBL4γ or DBL5β. This is a curious result as these recombinant domains were cloned from a laboratory parasite clone, A4. It implies that young children possess a degree of cross-reactive short-lived *Pf*EMP1-specific antibodies capable of recognizing individual domains.

It is interesting that not only did responses to DBL4γ correlate positively with individual responses to the intact A4-parasitized erythrocyte, but that these responses were also correlated with individual responses to an alternative A4 variant expressing a more heterogenous group of var genes on the erythrocyte surface and also to an unrelated laboratory clone, 3D7 and a clinical isolate, P1. That these correlations were not found with any other recombinant domain nor related to previous exposure as far as could be determined may suggest a cross-reactive or more conserved epitope maintained within the expressed DBL4γ protein.

Despite showing clear evidence of age and exposure dependent acquisition of domain specific responses and in addition demonstrating that those responses to DBL1α and DBL4γ correlate with antibody responses to the intact A4-parasitized erythrocyte, we were unable to convincing correlation with protection from clinical disease. By expressing each domain as a recombinant fusion protein in *E. coli*, it is likely that important conformational epitopes will not be accessible to the sera tested or equally that what is being recognized by sera are not important targets within the host Whether these antibodies are reacting to epitopes exposed by the process of cloning and expression within the bacterial system, epitopes not exposed as part of the full A4 *Pf*EMP1 molecule, or whether this reflects methodological limitations within the affinity purification process is presently unknown.

It is difficult to know how to interpret the apparent protective relationship between responses to DBL1α in parasite negative individuals in the higher transmission setting in this study. Previous studies in this area have shown an important effect of parasite positivity on the ability to detect protective responses against several blood stage antigens [28-30]. Given that in this case the effect is in the opposite direction, it must be considered that this is a chance finding and unlikely to be biologically meaningful.

Despite these limitations the data presented here have demonstrated the presence of antibodies in human sera to expressed domains of one variant of PfEMP1. An important component of investigating PfEMP1 as a vaccine candidate will involve determining what role antibodies have to both conserved and variable parts of this protein. The finding of potential cross-reactive responses to the domain DBL4γ in this work is potentially important and further work is needed to further clarify these responses.

## Competing interests

The authors declare that they have no competing interests.

## Authors' contributions

CLM, KM and CIN designed the study. CLM carried out the experimental work with technical assistance from ZC, RP and MK. TM and TNW set up the epidemiological framework and disease surveillance of the individuals involved in the study. CLM carried out data analysis and with KM and CIN wrote the paper. All authors read and approved the final manuscript.

## Supplementary Material

Additional file 1 **Mean OD values for positive standards on each plate**. Shown are the mean OD values obtained for pooled hyperimmune sera tested against each recombinant protein in turn. Each sera was tested in duplicate on each plate and each plate tested in full in duplicate. Click here for file

Additional file 2 **Range of OD values for 20 non-malaria exposed donors against each protein in turn**. Shown are the minimum, maximum, mean and median for the OD obtained for 20 non-malaria exposed sera, tested against each recombinant protein in turn. Each plate was tested in duplicate and each sample was tested once on each plate. Click here for file

Additional file 3 **Proportion of individuals in each age group recognizing parasite lines A4-40 cycle and 3D7 and the clinical isolate P1**. Sera from 140 individuals older than six months, was tested for reactivity against the parasite lines A4-40 cycle and 3D7 and the clinical isolate P1, a), b) and c) respectively, using flow cytometry. The proportion of individuals in each age category, with upper 95% confidence interval, scoring positive for antibody recognition are shown. Positivity was scored as defined in the text. The dark grey bars represent individuals resident in Chonyi and the light grey bars represent individuals resident in Ngerenya. Click here for file
