# Supplementary material for: Acquisition of naturally occurring antibody responses to recombinant protein domains of Plasmodium falciparum erythrocyte membrane protein 1
Source: Malar J. 2008 Aug 16;7:155. doi: 10.1186/1475-2875-7-155 (PMC2533674; doi:10.1186/1475-2875-7-155)
Supplement: Additional file 1 — Mean OD values for positive standards on each plate. Shown are the mean OD values obtained for pooled hyperimmune sera tested against each recombinant protein in turn. Each sera was tested in duplicate on each plate and each plate tested in full in duplicate. [file 1475-2875-7-155-S1.pdf]

Additional file 1

Mean OD values for positive standards on each plate

	DBL1α	DBL2β	CIDR1α	DBL4γ	DBL5β	MBP
**Plate 1**	1.1202	1.1397	0.9024	1.1683	1.3895	0.121
**Plate 2**	1.1108	1.1226	1.3671	1.8854	1.4034	0.1845
**Plate 3**	0.9144	1.2243	1.6563	1.5672	1.5047	0.215
**Plate 4**	1.5444	0.8522	1.5453	1.8717	1.4342	0.1857
**Plate 5**	0.7314	0.8761	1.2338	1.7454	1.3065	0.2185
**Plate 6**	1.2256	0.9853	1.3569	1.9043	1.2912	0.3255
**Plate 7**	0.9583	0.9005	1.1546	1.1245	1.2821	0.3856
**Plate 8**	0.9035	0.9477	1.2353	0.9987	1.4032	0.3595
**Plate 9**	0.7939	0.9692	1.0998	2.1162	1.0316	0.2921
**Plate10**	1.1977	0.9925	0.9330	1.9973	1.0716	0.2637
**Plate11**	0.9526	1.0287	1.0606	1.5867	1.2253	0.2421
**Plate12**	1.0383	1.0194	1.2984	2.4884	1.1616	0.3018
**Plate13**	1.1635	1.1951	1.4672	1.7228	0.9746	0.2095
**Plate14**	0.6623	0.9911	0.7065	1.6870	1.0387	0.1121
**Plate15**	1.1558	0.9503	1.3965	1.3228	1.1724	0.1734
