# Supplementary material for: Acquisition of naturally occurring antibody responses to recombinant protein domains of Plasmodium falciparum erythrocyte membrane protein 1
Source: Malar J. 2008 Aug 16;7:155. doi: 10.1186/1475-2875-7-155 (PMC2533674; doi:10.1186/1475-2875-7-155)
Supplement: Additional file 2 — Range of OD values for 20 non-malaria exposed donors against each protein in turn. Shown are the minimum, maximum, mean and median for the OD obtained for 20 non-malaria exposed sera, tested against each recombinant protein in turn. Each plate was tested in duplicate and each sample was tested once on each plate. [file 1475-2875-7-155-S2.pdf]

Additional file 2

Range of OD values for 20 non-malaria exposed donors against each protein in turn

	**Min**	**Max**	**Mean**	**Median**
**DBL1α**	0.008822	0.252871	0.123316	0.128204
**DBL2β**	0.114934	0.290416	0.229632	0.207405
**CIDR1α**	0.159987	0.642002	0.240013	0.306897
**DBL4ɣ**	0.053602	0.44863	0.116846	0.150796
**DBL5β**	0.010768	0.343139	0.174313	0.178731
