# Supplementary figures and images for: Acquisition of naturally occurring antibody responses to recombinant protein domains of Plasmodium falciparum erythrocyte membrane protein 1
Source: Malar J. 2008 Aug 16;7:155. doi: 10.1186/1475-2875-7-155 (PMC2533674; doi:10.1186/1475-2875-7-155)

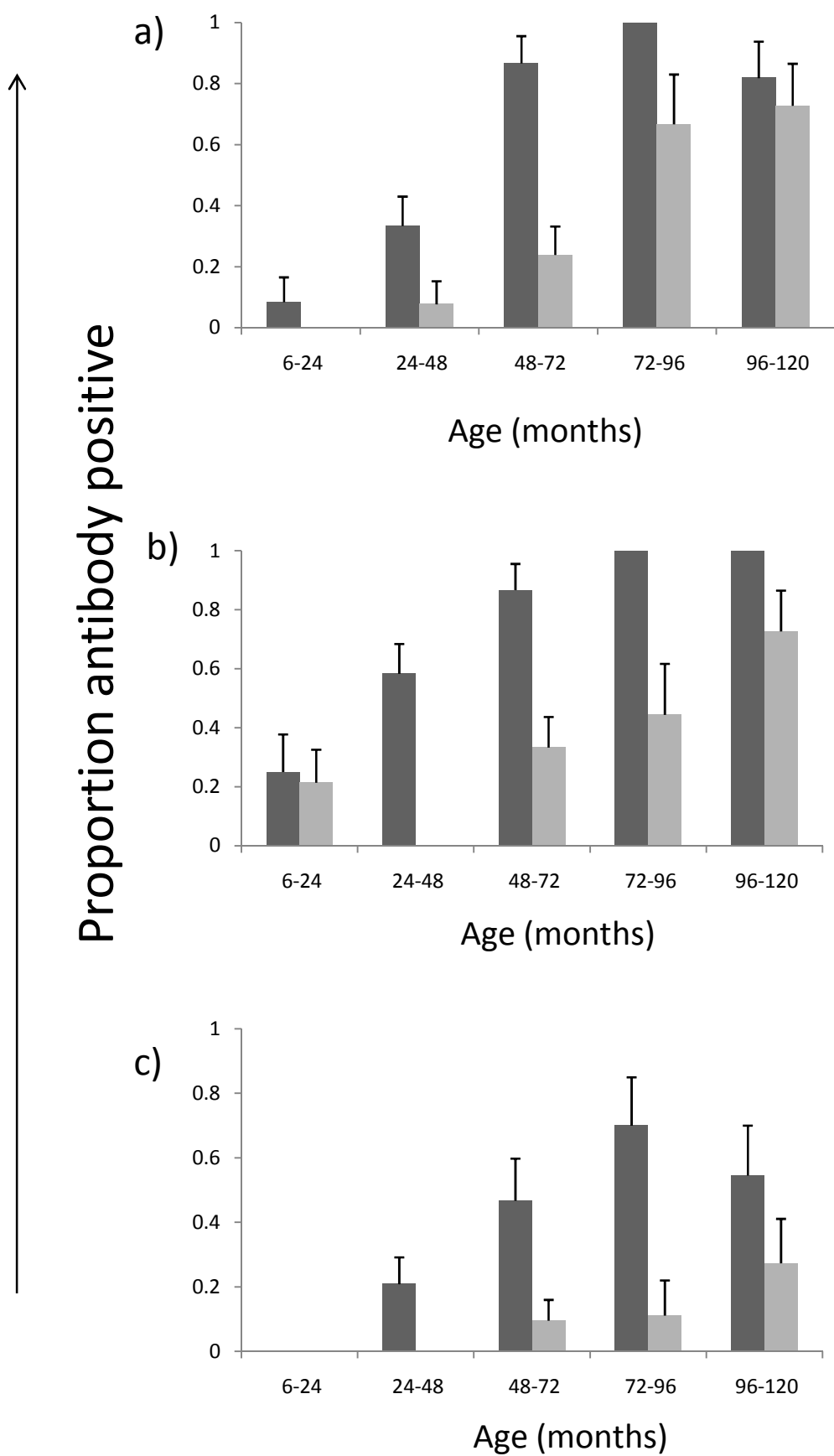

Supplement: Additional file 3 — Proportion of individuals in each age group recognizing parasite lines A4-40 cycle and 3D7 and the clinical isolate P1. Sera from 140 individuals older than six months, was tested for reactivity against the parasite lines A4-40 cycle and 3D7 and the clinical isolate P1, a), b) and c) respectively, using flow cytometry. The proportion of individuals in each age category, with upper 95% confidence interval, scoring positive for antibody recognition are shown. Positivity was scored as defined in the text. The dark grey bars represent individuals resident in Chonyi and the light grey bars represent individuals resident in Ngerenya. [file 1475-2875-7-155-S3.pdf]
